# Supplementary material for: Dimeric transport mechanism of human vitamin C transporter SVCT1
Source: Nat Commun. 2024 Jul 2;15:5569. doi: 10.1038/s41467-024-49899-2 (PMC11219872; doi:10.1038/s41467-024-49899-2)
Supplement: Supplementary file 1 — Supplementary Information [file 41467_2024_49899_MOESM1_ESM.pdf]

Supplementary Information for

Dimeric transport mechanism of human vitamin C transporter SVCT1

Takaaki A. Kobayashi[1], Hiroto Shimada[1†], Fumiya K. Sano[1], Yuzuru Itoh[1], Sawako Enoki[2], Yasushi Okada[2,3], Tsukasa Kusakizako[1*] & Osamu Nureki[1*]

[1] Department of Biological Sciences, Graduate School of Science, The University of Tokyo, Tokyo, Japan

[2] Department of Physics, Universal Biology Institute and International Research Center for Neurointelligence, The University of Tokyo, Tokyo, Japan

[3] Laboratory for Cell Polarity Regulation, RIKEN Center for Biosystems Dynamics Research, Osaka, Japan

[†] Present address: Chugai Pharmaceutical Co., Ltd., Tokyo, Japan

[*] Correspondence to: kusakizako@bs.s.u-tokyo.ac.jp (T.K.), nureki@bs.s.u-tokyo.ac.jp (O.N.)

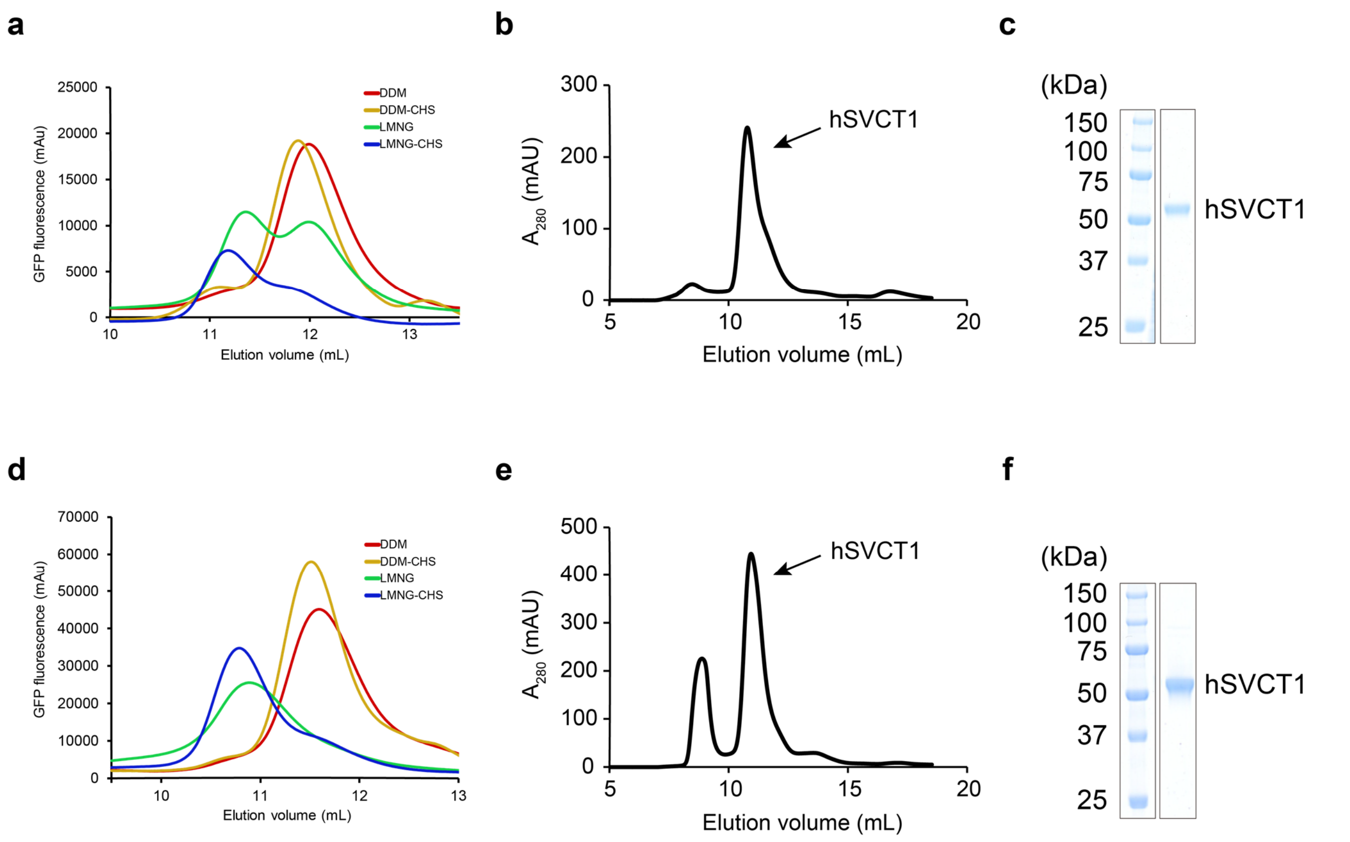

Supplementary Fig. 1 | Purification profiles of hSVCT1. a FSEC chromatograms of hSVCT1 with different detergents under sodium-containing conditions, detected by GFP fluorescence. Peak positions corresponding to the same oligomeric state exhibit a slight shift due to the different micelle sizes dependent on the detergent compositions. **b** SEC profile of hSVCT1 under the sodium-containing conditions. The elution fraction of hSVCT1 dimers is indicated. **c** SDS-PAGE analysis of the peak fraction corresponding to hSVCT1 dimers under the sodium-containing conditions. **d** FSEC chromatograms of hSVCT1 with different detergents under sodium-free conditions, detected by GFP fluorescence. **e** SEC profile of hSVCT1 under the sodium-free conditions. **f** SDS-PAGE analysis of the peak fraction corresponding to hSVCT1 dimers under the sodium-free conditions. Source data are provided as a Source Data file.

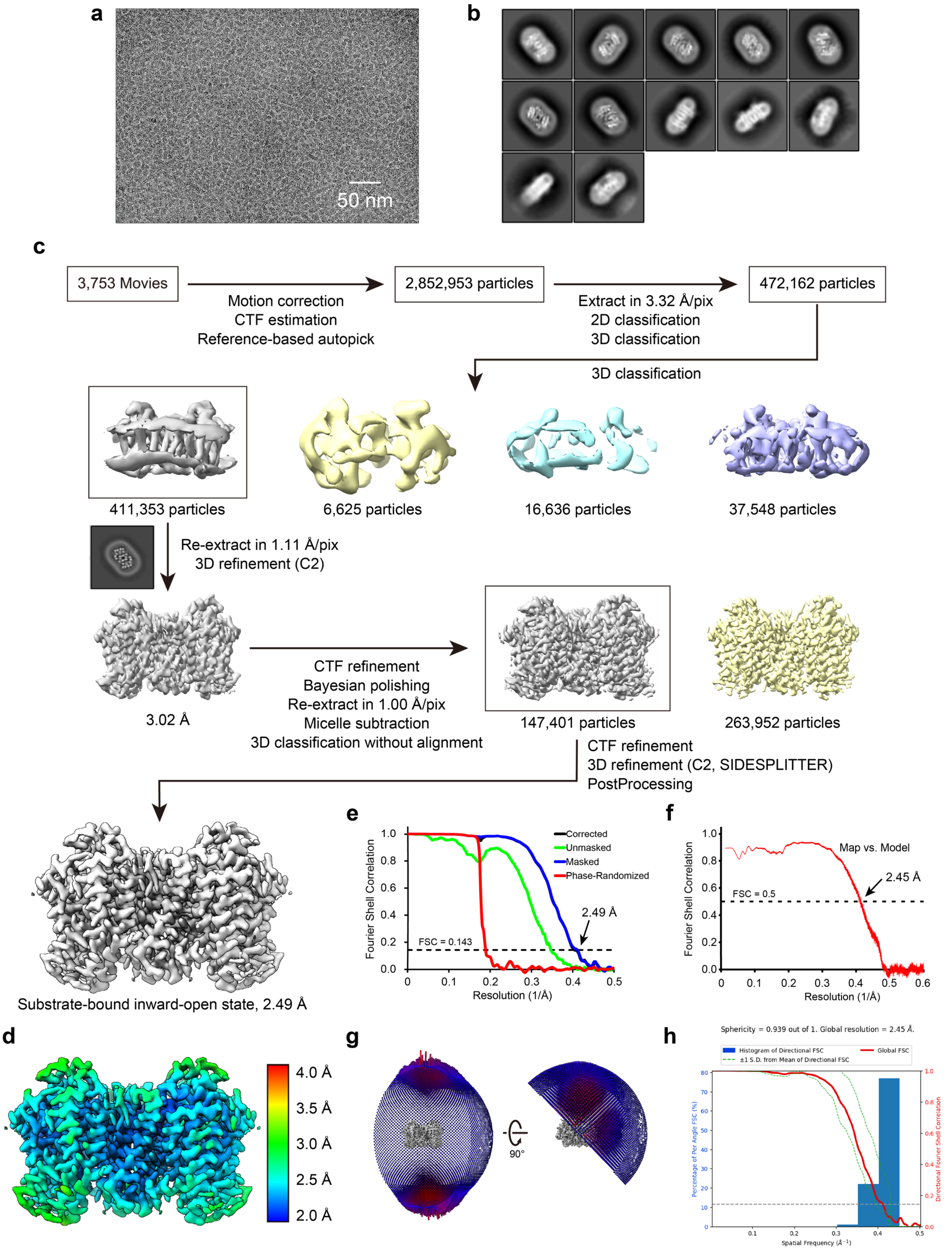
a
50 nm
b
c
3,753 Movies
Motion correction
CTF estimation
Reference-based autopick
2,852,953 particles
Extract in 3.32 Å/pix
2D classification
3D classification
472,162 particles
3D classification
411,353 particles
6,625 particles
16,636 particles
37,548 particles
Re-extract in 1.11 Å/pix
3D refinement (C2)
3.02 Å
CTF refinement
Bayesian polishing
Re-extract in 1.00 Å/pix
Micelle subtraction
3D classification without alignment
147,401 particles
263,952 particles
CTF refinement
3D refinement (C2, SIDESPLITTER)
PostProcessing
Substrate-bound inward-open state, 2.49 Å
e
Fourier Shell Correlation
Resolution (1/Å)
Corrected
Unmasked
Masked
Phase-Randomized
FSC = 0.143
2.49 Å
f
Fourier Shell Correlation
Resolution (1/Å)
Map vs. Model
FSC = 0.5
2.45 Å
d
4.0 Å
3.5 Å
3.0 Å
2.5 Å
2.0 Å
g
90°
h
Sphericity = 0.939 out of 1. Global resolution = 2.45 Å.
Histogram of Directional FSC
Global FSC
±1 S.D. from Mean of Directional FSC
Percentage of Per Angle FSC (%)
Directional Fourier Shell Correlation
Spatial Frequency (Å^{-1})

Supplementary Fig. 2 | Cryo-EM data processing of hSVCT1 in the substrate-bound inward-open state. a Representative cryo-EM micrograph of the hSVCT1 sample under sodium conditions supplemented with sodium ascorbate. **b** 2D class averages of particle images. **c** Flow chart of cryo-EM single particle analysis. **d** Local resolution map of the substrate-bound inward-open state. **e** Fourier Shell Correlation (FSC) curve of the final 3D reconstruction. **f** FSC curve for map-to-model fitting. **g** Angular distribution plot of the particles used for the final 3D reconstruction. **h** Directional 3DFSC plots calculated by the Remote 3DFSC Processing Server.

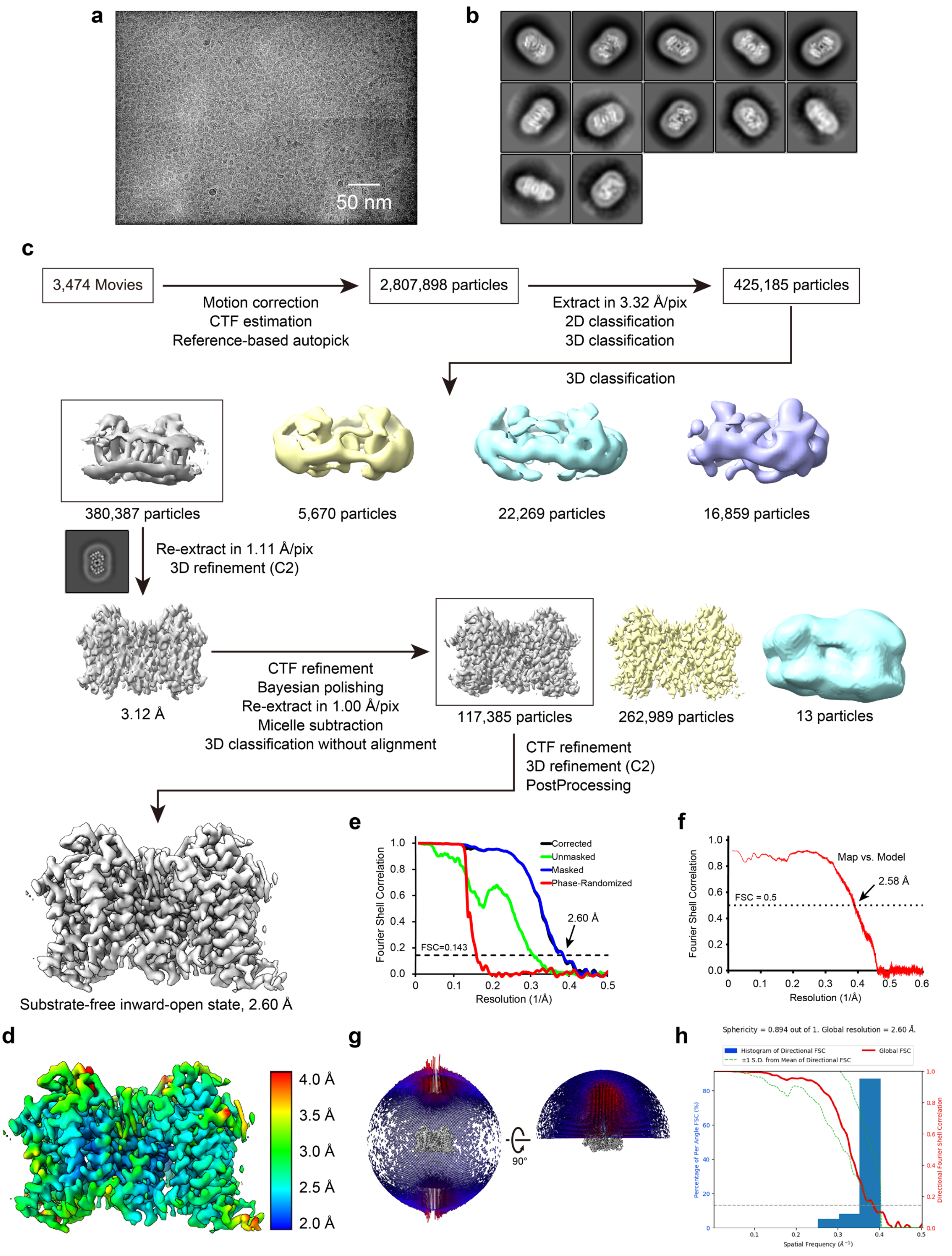
a
50 nm
b
c
3,474 Movies
Motion correction
CTF estimation
Reference-based autopick
2,807,898 particles
Extract in 3.32 Å/pix
2D classification
3D classification
425,185 particles
3D classification
380,387 particles
5,670 particles
22,269 particles
16,859 particles
Re-extract in 1.11 Å/pix
3D refinement (C2)
3.12 Å
CTF refinement
Bayesian polishing
Re-extract in 1.00 Å/pix
Micelle subtraction
3D classification without alignment
117,385 particles
262,989 particles
13 particles
CTF refinement
3D refinement (C2)
PostProcessing
Substrate-free inward-open state, 2.60 Å
e
Fourier Shell Correlation
Resolution (1/Å)
Corrected
Unmasked
Masked
Phase-Randomized
2.60 Å
FSC=0.143
f
Fourier Shell Correlation
Resolution (1/Å)
Map vs. Model
2.58 Å
FSC = 0.5
d
4.0 Å
3.5 Å
3.0 Å
2.5 Å
2.0 Å
g
90°
h
Sphericity = 0.894 out of 1. Global resolution = 2.60 Å.
Histogram of Directional FSC
±1 S.D. from Mean of Directional FSC
Global FSC
Percentage of Per Angle FSC (%)
Directional Fourier Shell Correlation
Spatial Frequency (Å⁻¹)

Supplementary Fig. 3 | Cryo-EM data processing of hSVCT1 in the substrate-free inward-open state. a Representative cryo-EM micrograph of the hSVCT1 sample under sodium conditions without sodium ascorbate. **b** 2D class averages of particle images. **c** Flow chart of cryo-EM single particle analysis. **d** Local resolution map of the substrate-free inward-open state. **e** Fourier Shell Correlation (FSC) curve of the final 3D reconstruction. **f** FSC curve for map-to-model fitting. **g** Angular distribution plot of the particles used for the final 3D reconstruction. **h** Directional 3DFSC plots calculated by the Remote 3DFSC Processing Server.

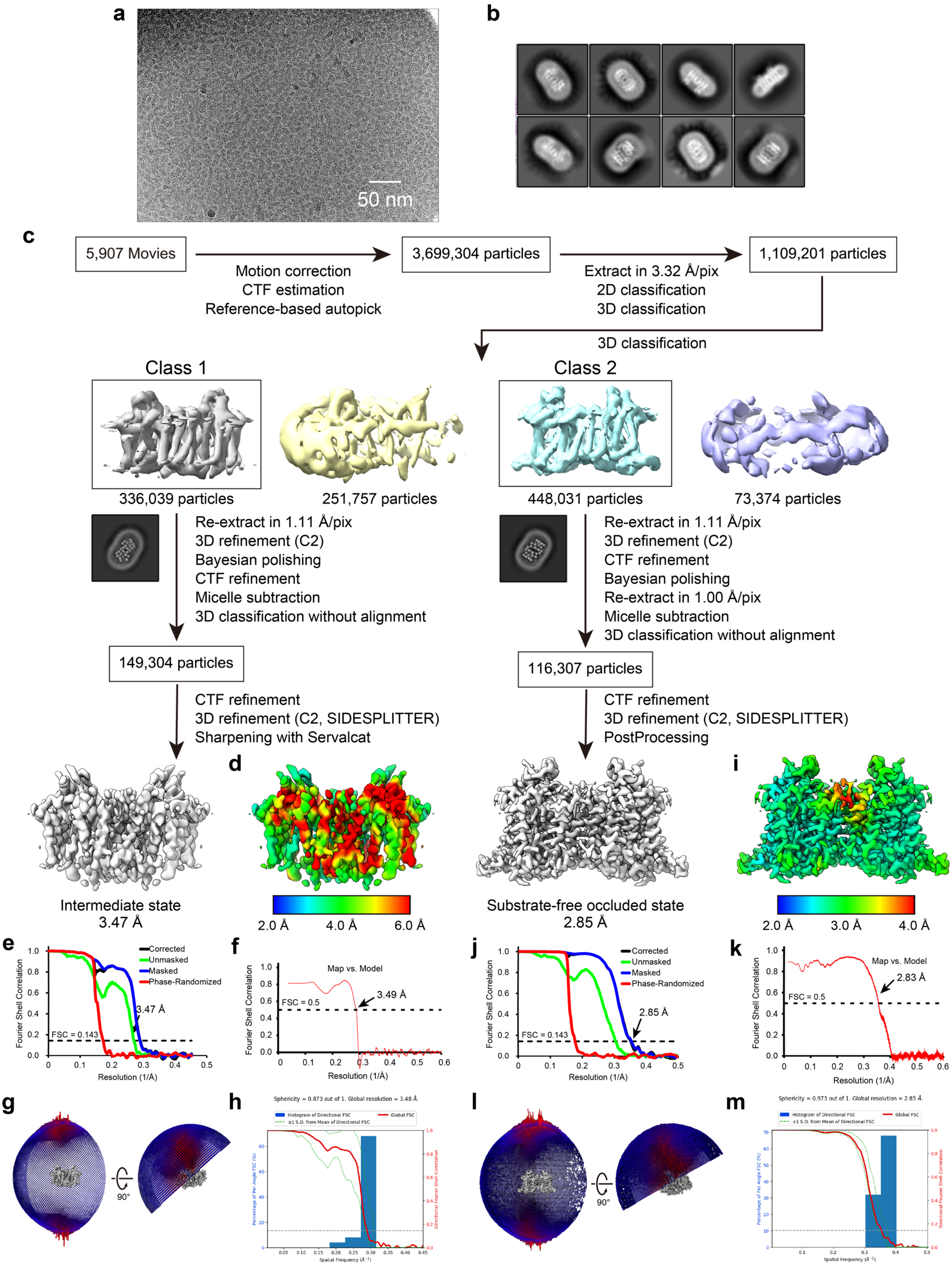

a
50 nm
b
c
5,907 Movies
Motion correction
CTF estimation
Reference-based autopick
3,699,304 particles
Extract in 3.32 Å/pix
2D classification
3D classification
1,109,201 particles
3D classification
Class 1
336,039 particles
251,757 particles
Class 2
448,031 particles
73,374 particles
Re-extract in 1.11 Å/pix
3D refinement (C2)
Bayesian polishing
CTF refinement
Micelle subtraction
3D classification without alignment
Re-extract in 1.11 Å/pix
3D refinement (C2)
CTF refinement
Bayesian polishing
Re-extract in 1.00 Å/pix
Micelle subtraction
3D classification without alignment
149,304 particles
116,307 particles
CTF refinement
3D refinement (C2, SIDESPLITTER)
Sharpening with Servalcat
CTF refinement
3D refinement (C2, SIDESPLITTER)
PostProcessing
d
i
Intermediate state
3.47 Å
2.0 Å
4.0 Å
6.0 Å
Substrate-free occluded state
2.85 Å
2.0 Å
3.0 Å
4.0 Å
e
Corrected
Unmasked
Masked
Phase-Randomized
FSC = 0.143
3.47 Å
Fourier Shell Correlation
Resolution (1/Å)
f
Map vs. Model
3.49 Å
FSC = 0.5
Fourier Shell Correlation
Resolution (1/Å)
j
Corrected
Unmasked
Masked
Phase-Randomized
FSC = 0.143
2.85 Å
Fourier Shell Correlation
Resolution (1/Å)
k
Map vs. Model
2.83 Å
FSC = 0.5
Fourier Shell Correlation
Resolution (1/Å)
g
90°
h
Sphericity = 0.873 out of 1. Global resolution = 3.48 Å.
l
90°
m
Sphericity = 0.973 out of 1. Global resolution = 2.85 Å.

Supplementary Fig. 4 | Cryo-EM data processing of hSVCT1 under the sodium-free conditions. a Representative cryo-EM micrograph of the hSVCT1 sample under sodium-free conditions without potassium ascorbate. **b** 2D class averages of particle images. **c** Flow chart of cryo-EM single particle analysis. The two boxed 3D classes correspond to “class 1” and “class 2” in the text. **d** Local resolution map of the intermediate state. **e** Fourier Shell Correlation (FSC) curve of the final 3D reconstruction of the intermediate state. **f** FSC curve for map-to-model fitting of the intermediate state. **g** Angular distribution plot of the particles used for the final 3D reconstruction of the intermediate state. **h** Directional 3DFSC plots of the intermediate state calculated by the Remote 3DFSC Processing Server. **i** Local resolution map of the substrate-free occluded state. **j** FSC curve of the final 3D reconstruction of the substrate-free occluded state. **k** FSC curve for map-to-model fitting of the substrate-free occluded state. **l** Angular distribution plot of the particles used for the final 3D reconstruction of the substrate-free occluded state. **m** Directional 3DFSC plots of the substrate-free occluded state calculated by the Remote 3DFSC Processing Server.

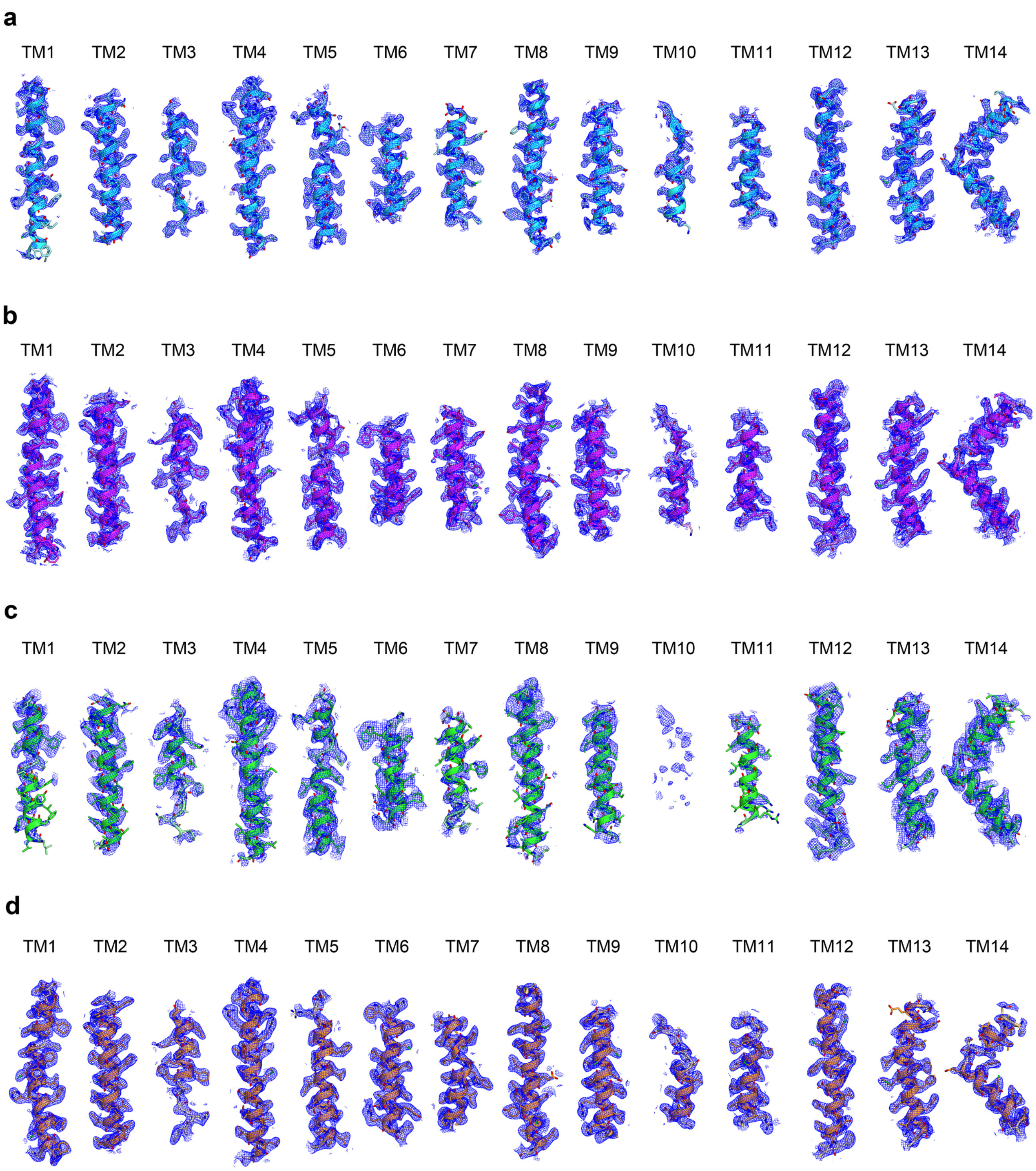

Supplementary Fig. 5 | Fit of structural models to density maps. All transmembrane helices are shown for (**a**) substrate-bound inward-open state, (**b**) substrate-free inward-open state, (**c**) intermediate state, and (**d**) substrate-free occluded state. TM10 in the intermediate state is disordered and not modelled.

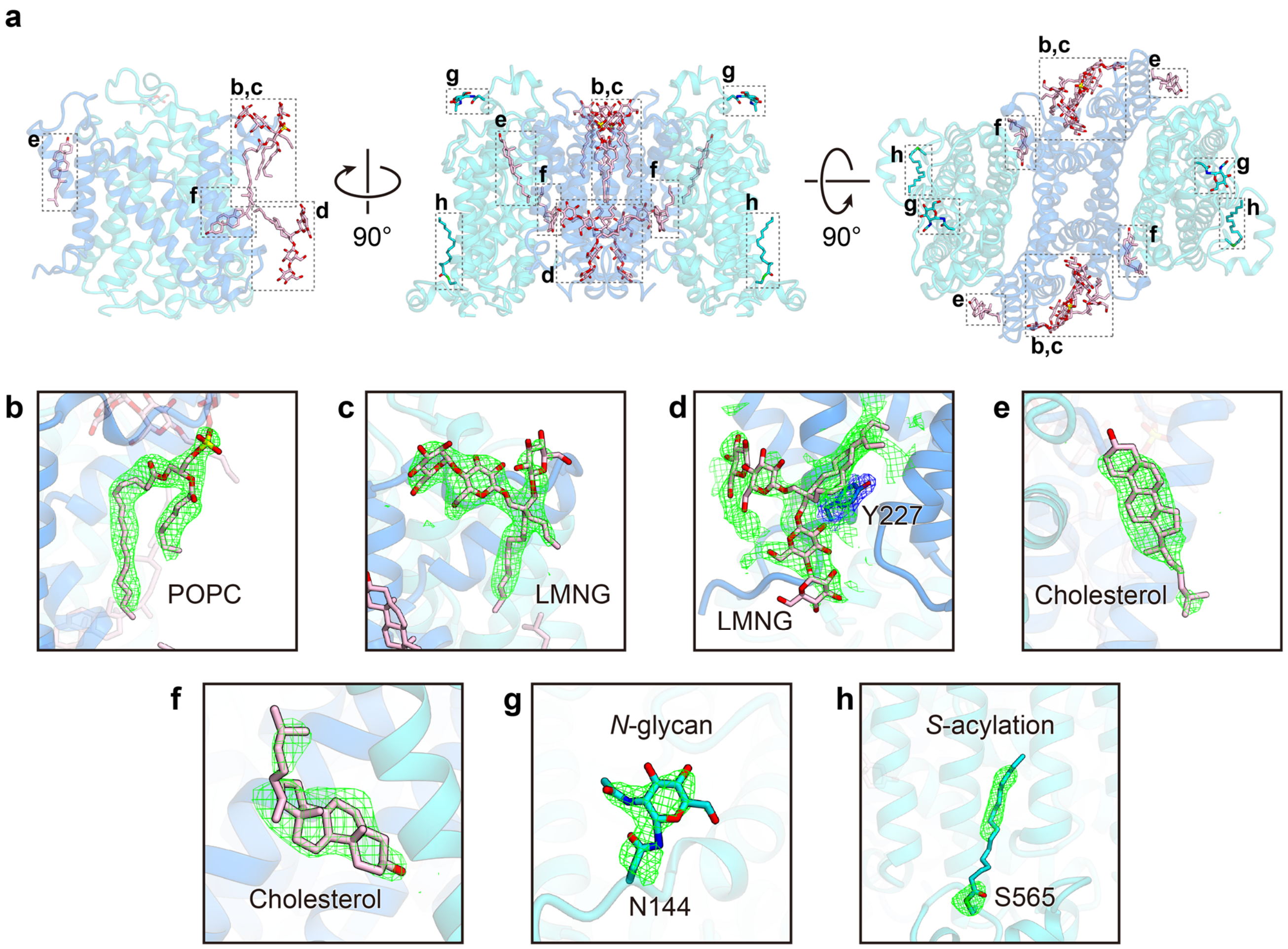

Supplementary Fig. 6 | Lipids, detergent molecules and posttranscriptional modifications observed in the inward-open structure. a hSVCT1 inward-open structure, with lipids and modifications shown as sticks. Dashed rectangles represent the positions for each molecule in the subsequent close-up views. **b**–**h** Close-up views of lipids and modifications in the inward-open structure. (**g**) *N*-acetylglucosamine and (**h**) *S*-palmitoyl cysteine, modeled for posttranscriptional modifications. The green mesh represents the F_o-F_c omit map for each molecule.

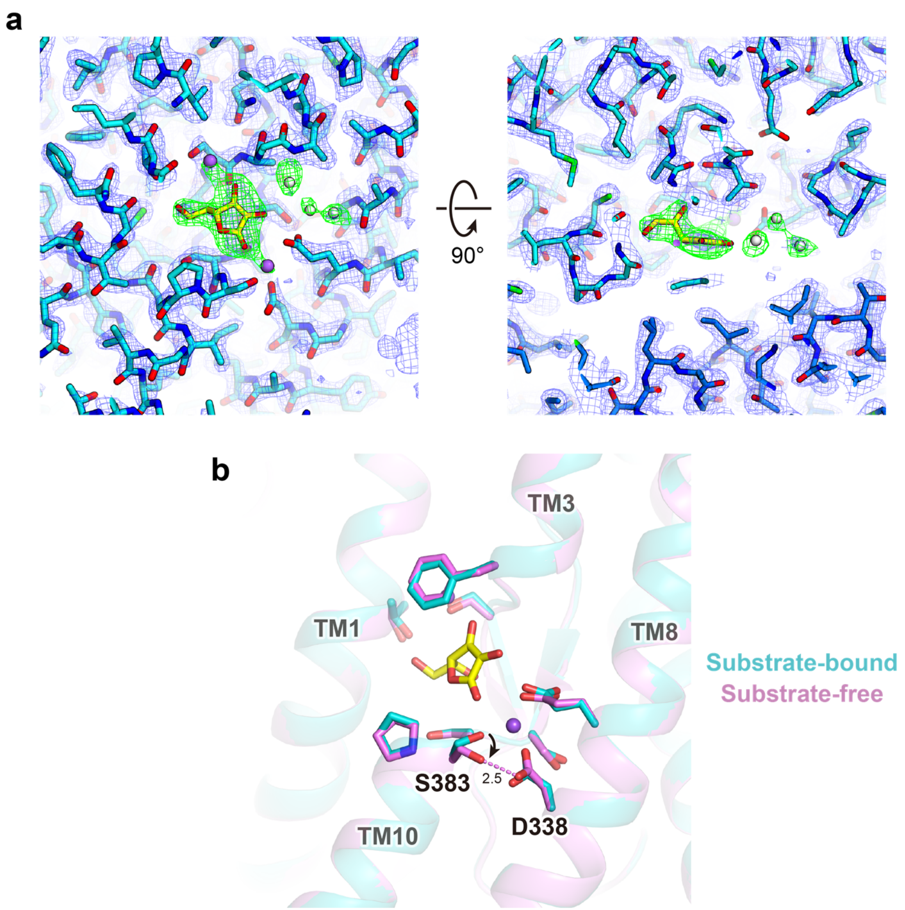

Supplementary Fig. 7 | Observation and comparison of inward-open structures at the substrate binding pocket. **a** Density map of the substrate binding pocket viewed from (**left**) the gate domain of the same protomer and (**right**) the extracellular side. Blue and green meshes correspond to the observed density and the F_o-F_c omit map for ligands, respectively (both contoured at 3.0σ). **b** Comparison of residues at the substrate binding pocket between the substrate-bound (colored cyan) and substrate-free (colored violet) inward-open states, aligned by the monomeric core domains. The arrow represents the flipping of the Ser383 sidechain and the dashed line represents the interaction distance (Å) between Asp338 and Ser383 in the substrate-free inward-open state.

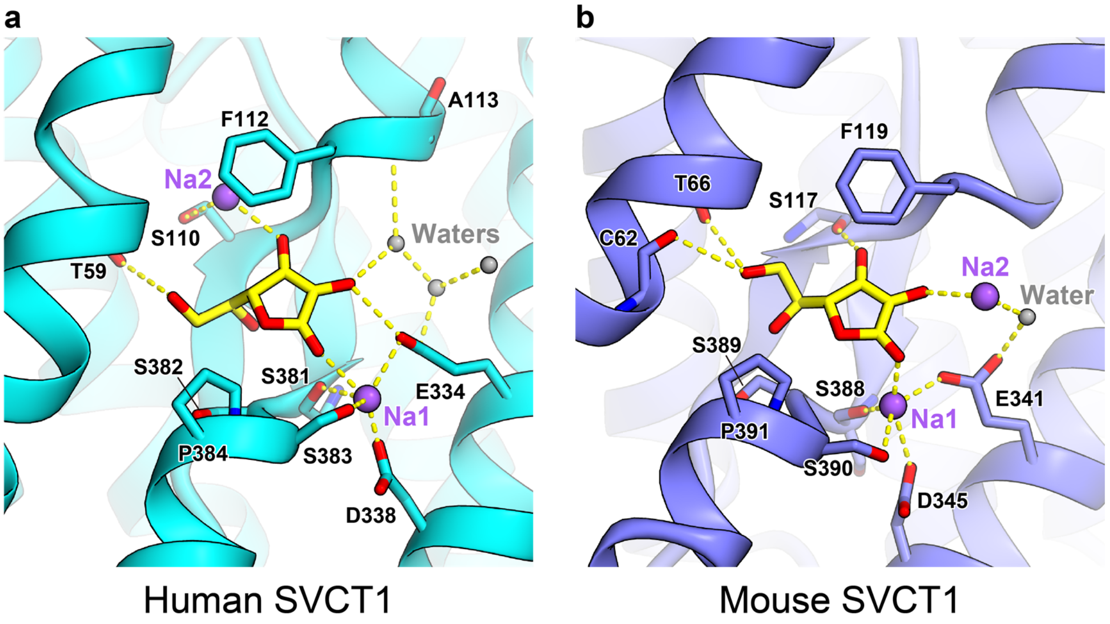

Supplementary Fig. 8 | Comparison of substrate recognition mechanisms between human and mouse SVCT1. Coordination manners of vitamin C, sodium, and water molecules in the (**a**) human SVCT1 structure (this study) and the (**b**) mouse SVCT1 structure (PDB 7YTW).

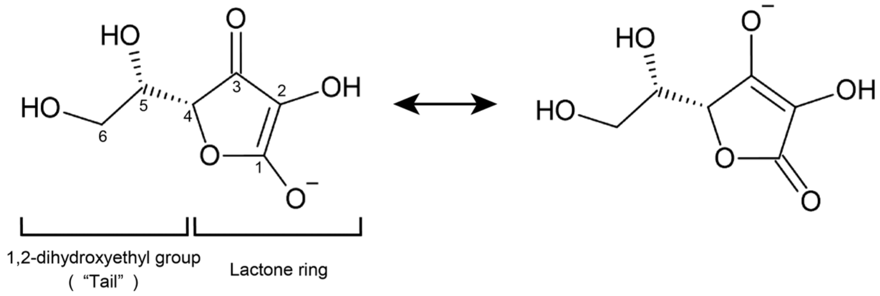

Supplementary Fig. 9 | Canonical forms of L-ascorbic acid in resonance between two monoanions under neutral pH conditions. The numbering of the carbon atoms and the names of the structural components of the molecule referred to in the text are shown on the left.

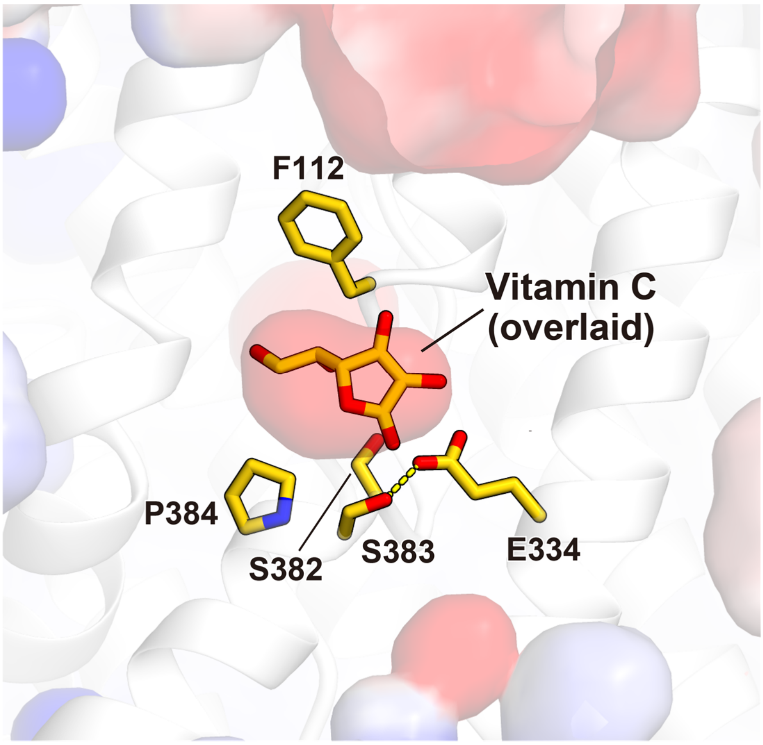

Supplementary Fig. 10 | Overlay of a vitamin C molecule on the occluded substrate binding pocket in the hSVCT1 structure. The surface representation is colored by the electrostatic potential, with transparency. A manually modeled vitamin C molecule cannot be positioned within the occluded pocket without protruding.

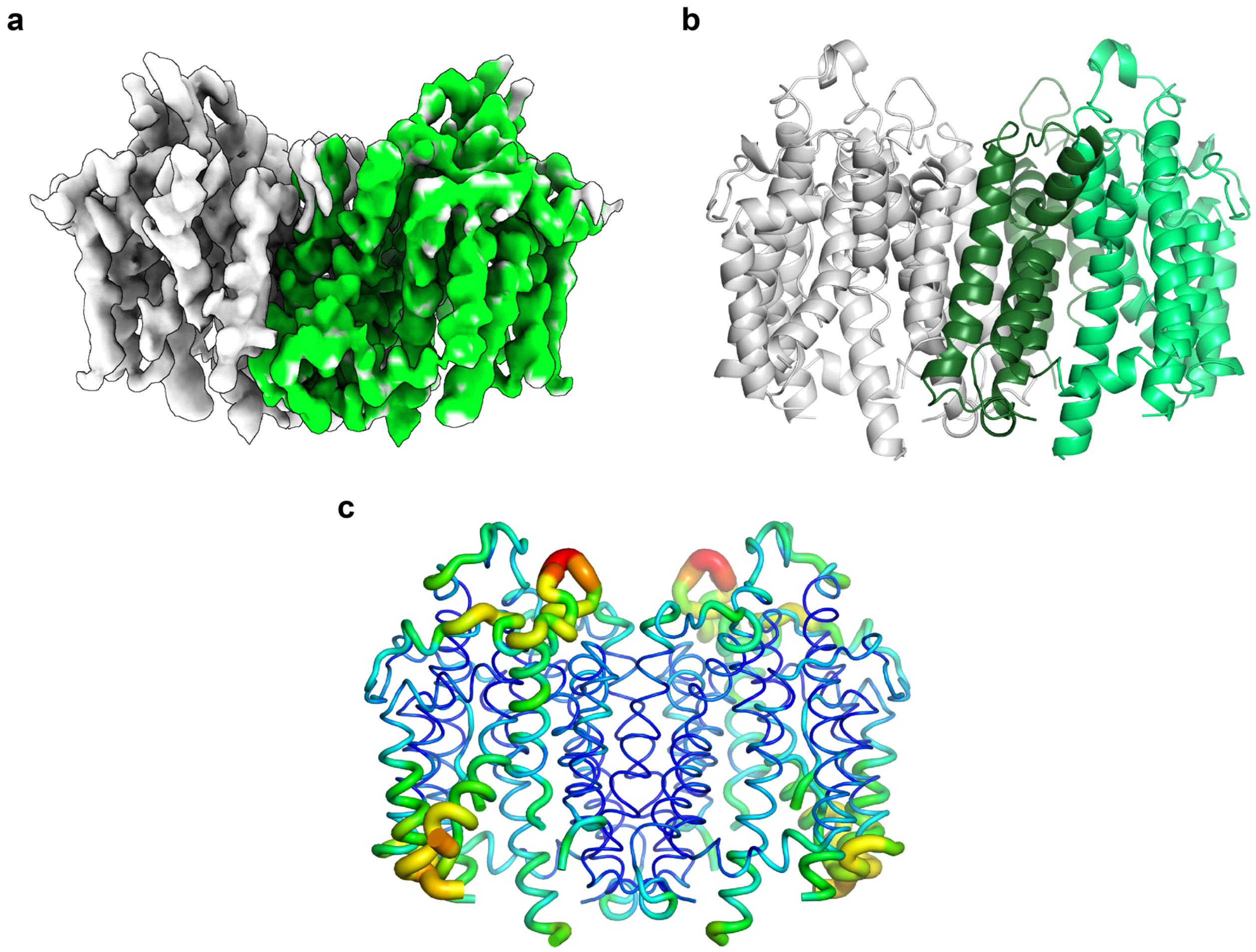

Supplementary Fig. 11 | Structure of hSVCT1 in the putative intermediate state between inward-open and substrate-free occluded states. a Cryo-EM map of the hSVCT1 homodimer in the intermediate state, with densities corresponding to each protomer colored green and grey, respectively. **b** Overall structure of hSVCT1 in the intermediate state. The core and gate domains of a protomer are colored neon green and dark green, respectively. **c** *B*-factor putty representation of the intermediate structure. The cytosolic region of the core domain, which is disordered in the obtained density map, is displayed as thick tubes and indicates the high mobility of the structure.

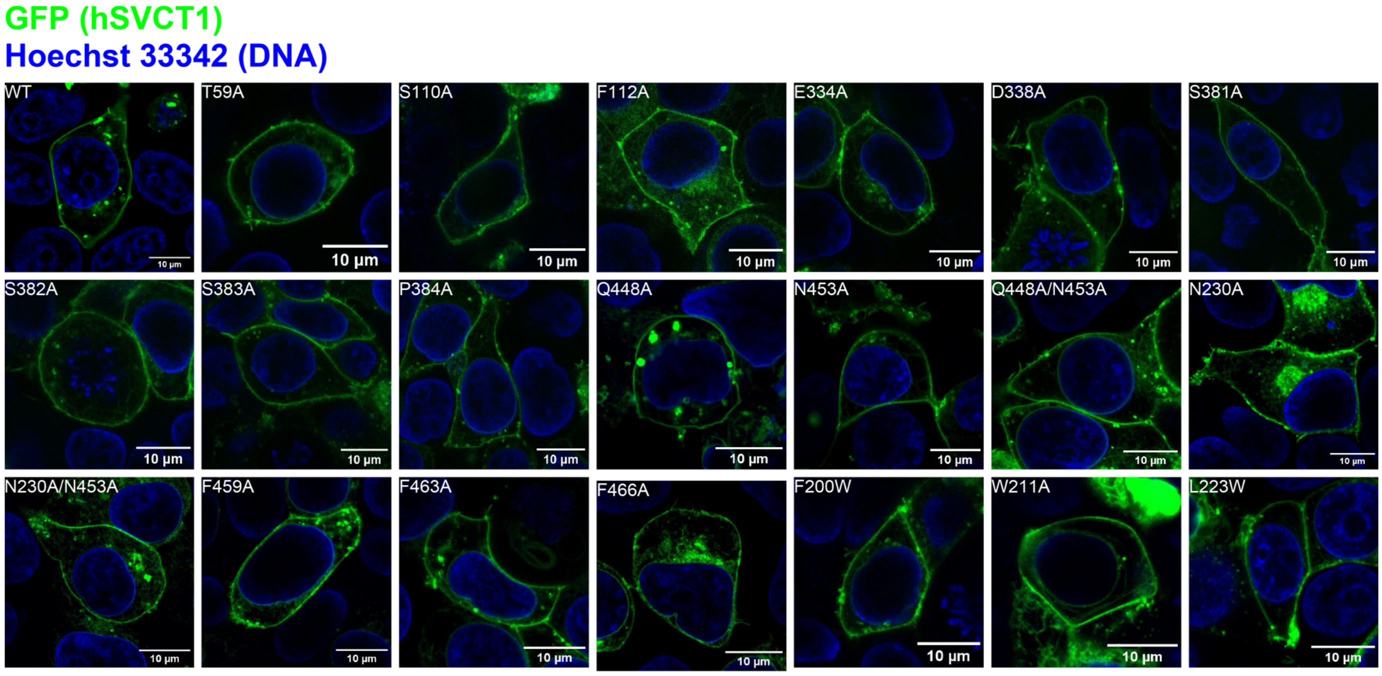

Supplementary Fig. 12 | Plasma membrane expression of hSVCT1 mutants. Representative fluorescence images of HEK293T cells expressing C-terminally GFP-tagged hSVCT1 mutants. DNA was stained with Hoechst 33342. The observations were repeated at least three times independently with similar results.

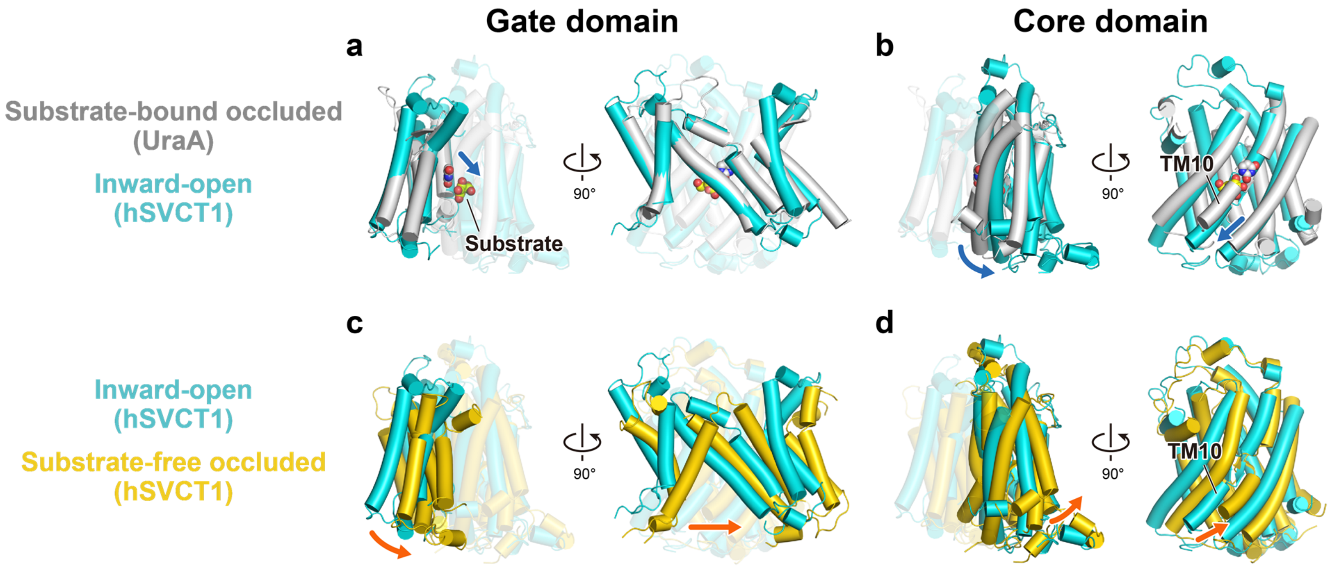

Supplementary Fig. 13 | Domain-based dimeric comparisons of substrate-bound occluded, inward-open, and substrate-free occluded structures. a-b Comparisons of (**a**) the gate domain and (**b**) the core domain between substrate-bound occluded UraA (grey) and inward-open hSVCT1 (cyan), superimposed by the dimeric gate domains. **c-d** Comparison of (**c**) the gate domain and (**d**) the core domain between inward-open hSVCT1 (cyan) and substrate-free occluded hSVCT1 (yellow), superimposed by the dimeric core domains. Arrows indicate the relative movements of domains and the substrate. For simplicity, one protomer is not shown in each figure.

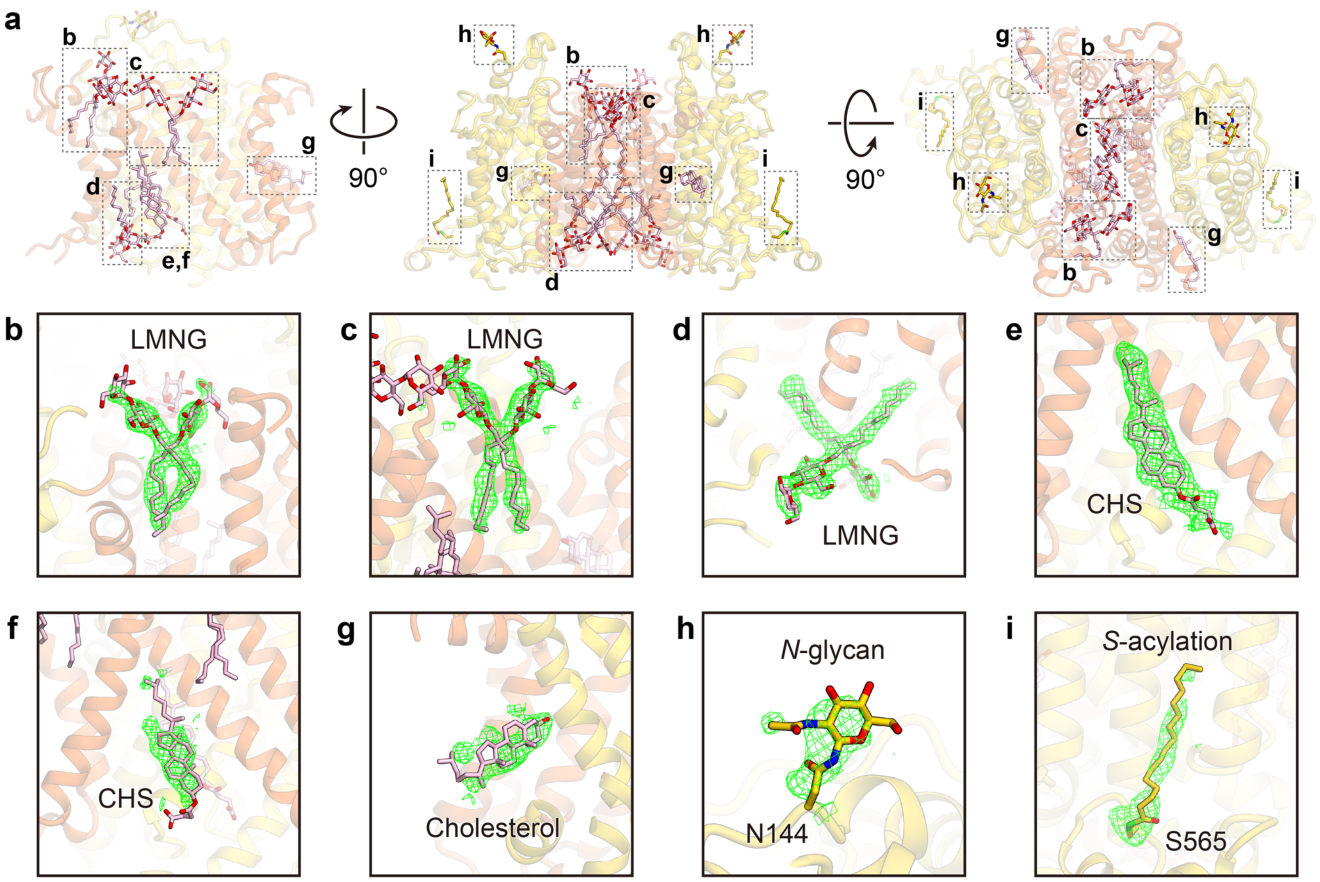

Supplementary Fig. 14 | Lipids, detergent molecules and posttranscriptional modifications observed in the substrate-free occluded hSVCT1 structure. a hSVCT1 substrate-free occluded structure, with lipids and modifications shown as sticks. Dashed rectangles represent the positions of each molecule in the subsequent close-up views. **b-i** Close-up views of lipids and modifications in the substrate-free occluded structure. (**h**) *N*-acetylglucosamine and (**i**) *S*-palmitoyl cysteine, modeled for posttranscriptional modifications. The green mesh represents the F_o-F_c omit map for each molecule.

Supplementary Table 1 | Cryo-EM data collection, refinement and validation statistics.

	hSVCT1 Substrate-bound inward-open state (EMDB-36201) (PDB 8JEW)	hSVCT1 Substrate-free inward-open state (EMDB-36204) (PDB 8JEZ)	hSVCT1 Intermediate state (EMDB-36205) (PDB 8JF0)	hSVCT1 Substrate-free occluded state (EMDB-36206) (PDB 8JF1)
**Data collection and processing**				
Magnification	105,000	105,000	105,000	
Voltage (kV)	300	300	300	
Electron exposure (e^{-}/Å^{2})	49.7	50.0	50.9	
Defocus range (μm)	-0.8 to -1.6	-0.8 to -1.6	-0.8 to -1.6	
Pixel size (Å)	0.83	0.83	0.83	
Symmetry imposed	C2	C2	C2	C2
Initial particle images (no.)	2,852,953	2,807,898	3,699,304	
Final particle images (no.)	147,401	117,385	149,304	116,307
Map resolution (Å)	2.49	2.60	3.47	2.85
FSC threshold	0.143	0.143	0.143	0.143
**Refinement**				
Initial model used (PDB code)	AlphaFold	8JEW	8JEZ	8JEW
Model resolution (Å)	2.45	2.58	3.49	2.83
FSC threshold	0.5	0.5	0.5	0.5
Model composition				
Non-hydrogen atoms	8,284	8,282	6,578	8,442
Protein residues	1,024	1,026	872	1,026
Ligands	26	16	0	20
B factors (Å^{2})				
Protein	70.70	68.99	151.49	71.73
Ligand	139.56	134.59	n/a	117.98
R.m.s. deviations				
Bond lengths (Å)	0.0148	0.0158	0.0158	0.0149
Bond angles (°)	1.84	1.85	1.73	1.88
Validation				
MolProbity score	1.51	1.38	1.93	1.31
Clashscore	6.25	6.13	9.31	4.11
Poor rotamers (%)	0.48	0.48	0.28	0.71
Ramachandran plot				
Favored (%)	97.04	97.83	93.22	97.43
Allowed (%)	2.96	2.17	6.78	2.57
Disallowed (%)	0	0	0	0
