# Supplementary material for: Dimeric transport mechanism of human vitamin C transporter SVCT1
Source: Nat Commun. 2024 Jul 2;15:5569. doi: 10.1038/s41467-024-49899-2 (PMC11219872; doi:10.1038/s41467-024-49899-2)
Supplement: Supplementary file 3 — Description of Additional Supplementary Files [file 41467_2024_49899_MOESM3_ESM.pdf]

Description of Additional Supplementary Files:

Supplementary Movie 1: Structural transitions of hSVCT1 in the transport cycle.
